# Excitotoxic lesions in the central nucleus of the amygdala attenuate stress-induced anxiety behavior

**DOI:** 10.3389/fnbeh.2013.00032

**Published:** 2013-04-19

**Authors:** Ana P. Ventura-Silva, António Melo, Ana C. Ferreira, Miguel M. Carvalho, Filipa L. Campos, Nuno Sousa, José M. Pêgo

**Affiliations:** ^1^Life and Health Sciences Research Institute (ICVS), School of Health Sciences, University of MinhoBraga, Portugal; ^2^ICVS/3B's - PT Government Associate LaboratoryBraga/Guimarães, Portugal

**Keywords:** BNST, central amygdala, anxiety, excitotoxic lesion

## Abstract

The extended amygdala, composed by the amygdaloid nuclei and the bed nucleus of the stria terminalis (BNST), plays a critical role in anxiety behavior. In particular, the link between the central nucleus of the amygdala (CeA) and the BNST seems to be critical to the formation of anxiety-like behavior. Chronic unpredictable stress (CUS) exposure is recognized as a validated animal model of anxiety and is known to trigger significant morphofunctional changes in the extended amygdala. Quite surprisingly, no study has ever analyzed the role of the CeA in the onset of stress-induced anxiety and fear conditioning behaviors; thus, in the present study we induced a bilateral excitotoxic lesion in the CeA of rats that were subsequently exposed to a chronic stress protocol. Data shows that the lesion in the CeA induces different results in anxiety and fear-behaviors. More specifically, lesioned animals display attenuation of the stress response and of stress-induced anxiety-like behavior measured in the elevated-plus maze (EPM) when compared with stressed animals with sham lesions. This attenuation was paralleled by a decrease of stress-induced corticosterone levels. In contrast, we did not observe any significant effect of the lesion in the acoustic startle paradigm. As expected, lesion of the CeA precluded the appearance of fear behavior in a fear-potentiated startle paradigm in both non-stressed and stressed rats. These results confirm the implication of the CeA in fear conditioning behavior and unravel the relevance of this brain region in the regulation of the HPA axis activity and in the onset of anxiety behavior triggered by stress.

## Introduction

Anxiety disorders are very prevalent (Kessler et al., 2009). Anxiety is characterized by a sensation of discomfort and apprehension in response to unconditioned diffuse cues (Koch, 1999). It is well-established that exposure to chronic stress is a triggering factor for development of anxiety. Stress induces several alterations in the central nervous system, with particular relevance to areas in the limbic system that regulate the stress response and emotional behavior. It has been shown that different models of stress can alter dendritic and synaptic plasticity with contrasting patterns, namely atrophy in the prefrontal cortex (Cerqueira et al., 2005, 2007; Dias-Ferreira et al., 2009) and hippocampus (Bessa et al., 2009) and hypertrophy in the bed nucleus of the stria terminalis (BNST; Pêgo et al., 2008) and the amygdala (Vyas et al., 2002, 2003; McEwen et al., 2012).

Among the limbic structures, the extended amygdala, which comprises, among other areas, the BNST and the central nucleus of the amygdala (CeA) (Alheid et al., 1998) plays a major role in the modulation of anxiety behavior. In particular, CeA is an output area of the amygdala and is involved in both fear and anxiety behaviors (Walker et al., 2009; Davis et al., 2010). Although the CeA does not have strong direct projections to the hypothalamus (Moga and Saper, 1994; Prewitt and Herman, 1998), its activation will lead to the stimulation of hypothalamic nuclei and areas that are responsible for fear and stress responses (Beaulieu et al., 1987; Shepard et al., 2006). This stimulation occurs largely through a massive projection from CeA to the BNST, a region that in turn projects densely to the paraventricular nucleus of the hypothalamus (PVN) (Dong et al., 2001). Indeed, it is presently recognized that the BNST acts as a relay station between upper limbic areas and the PVN, playing a fundamental role in the modulation of the stress response and anxiety behaviors (Herman et al., 2005; Choi et al., 2007).

The connection between the CeA and the BNST occurs through the stria terminalis, a bundle of projection fibers that include GABAergic neurons co-expressing peptides such as corticotrophin releasing factor (CRF) or enkephalin (Veinante et al., 1997; Day et al., 1999). Of relevance, CRF is highly expressed in the CeA, with this region being one major extra-hypothalamic source of this peptide. Due to the role of CRF in stress response, it has been proposed that neurons expressing CRF in the CeA are involved in stress related anxiety and fear behavior (Davis, 1992; Makino et al., 1995). It was shown that exposure to stress induces increased expression of CRF in several brain regions, including the extended amygdala (Kalin et al., 1994; Makino et al., 1994; Cook, 2004; Shepard et al., 2006). Previous reports have shown that a lesion in CeA is able to alter the basal levels of CRF in the PVN but interestingly not after stress (Prewitt and Herman, 1997). Quite surprisingly, to the best of our knowledge, there are no reports about the consequences of lesions in the CeA in the development of anxiety-like behavior in a rodent model of chronic stress. To further understand the role that this area plays in anxiety and the fear-potentiated startle behavior we assessed how excitotoxic lesions of CeA affect the development of anxiety induced by a chronic unpredictable stress (CUS) paradigm.

## Materials and methods

### Animals and treatments

Animal experiments were conducted in accordance with the European Communities Council Directive (86/609/EEC) and the NIH guidelines on animal care and experimentation. All experiments were approved by the Animal Ethics Committee of the Portuguese National Veterinary Directorate.

Adult male Wistar rats (Charles Rivers Laboratories, Barcelona, Spain) were housed in groups of 2 per cage under standard laboratory conditions (temperature 22°C; artificial light/dark cycle of 12/12 h; lights on at 8 a.m) and with *ad-libitum* access to commercial chow and water.

### Surgery

Forty male rats (8 weeks old) were submitted to stereotaxic surgery under ketamine/medetomidine anaesthesia. The animals were randomly distributed to one of two groups. A group of animals was injected with phosphate buffer solution (PBS) (*n* = 20) and other (*n* = 20) with ibotenic acid (Sigma-Aldrich, St. Louis, Misouri, USA) in the central amygdala (−2.2 mm from bregma, 4.2 mm from midline, 7.0 mm from brain surface). Ibotenic acid (10 mg/ml) was injected at a rate of 0.05 μl/min for a total volume of 0.2 μl.

After the surgery animals were given 1 week to rest and then were subdivided into four groups: Control-Sham (Cont-Sham; *n* = 10 randomly chosen from animals injected with vehicle); control-lesion (Cont-Lesion; *n* = 10 randomly chosen from animals that received ibotenic acid); a CUS-Sham (*n* = 10 corresponding to animals injected with vehicle) and CUS-Lesion (*n* = 10 from the animals injected with ibotenic acid).

### Stress protocol

Stress protocol started when the animals were 9 weeks of age, and it lasted 28 days. Animals were exposed to one different stressor per day (30 min/day) of one of the following aversive stimuli: immersion in cold water (18°C), vibration of the home cage, restraining, overcrowding, and exposure to a hot air stream (Cerqueira et al., 2007). The stressors were scheduled in a random order for the duration of the experiment. Control animals were handled on a daily basis over the 4 weeks. Weekly body weights and post-mortem weight of adrenals and thymus were recorded as indicator of the efficacy of the stress protocol (Table 1).

**Table 1:** **Biometric markers revealed that the CUS protocol decreased body-weight gain**.

	**Control-sham**	**Control-lesion**	**CUS-sham**	**CUS-lesion**		**Significance**
Body weight gain (g)	98.2 ± 2.5	106.2 ± 2.4	83.8 ± 3.6	90.3 ± 2.9	*F*_(1, 37)_ = 3.48	*P* < 0.05
Thymus weight (gr/BW)	0.46 ± 0.02	0.47 ± 0.01	0.37 ± 0.01	0.38 ± 0.01	*F*_(1, 37)_ = 4.45	*P* < 0.110
Adrenal weight (gr/BW)	0.44 ± 0.01	0.46 ± 0.01	0.52 ± 0.01	0.48 ± 0.01	*F*_(1, 37)_ = 1.35	*P* < 0.32

Significance corresponds to the ANOVA between groups. Data presented as Mean ± SEM.

### Behavioral tests

After the end of the stress exposure a behavioral evaluation was performed to assess anxiety-like behavior [elevated-plus maze (EPM) and acoustic startle], fear conditioning (fear-potentiated startle) and locomotor activity (open field). Behavioral tests were performed in the following order to minimize the effects each test could have in the following test: EPM, open field, acoustic startle and fear-potentiated startle. The behavioral tests started 24 h after the last stressor was applied. The acoustic startle and fear-potentiated startle were spaced 1 week in which the animals were still submitted to the chronic stress protocol.

### Elevated plus maze

Animals were tested over 5 min in a black polypropylene “plus”-shaped maze (ENV-560, MedAssociates Inc, St. Albans, VT 05478) at a height of 72 cm above the floor (EPM). The maze consisted of two facing open arms (50.8 × 10.2 cm) and two closed arms (50.8 × 10.2 × 40.6 cm). Testing was performed under bright white light. The time spent in the open arms, junction area and closed arms, as well as the number of entrances and explorations in each section were recorded using a system of infrared photobeams, the crossings of which were monitored by computer. The times spent in each of the compartments of the EPM are presented as percentage of the total duration of the trial.

### Acoustic startle

Startle reflex (ASR) was measured in startle response apparatuses (SR-LAB, San Diego Instruments, San Diego, CA, USA), each consisting of a non-restrictive Plexiglas cylinder (inner diameter 8.8 cm, length 22.2 cm), mounted on a Plexiglas platform and placed in a ventilated, sound-attenuated chamber. Animals were habituated to the apparatus (5 min daily) for 2 days before actual testing. Cylinder movements were detected and measured by a piezoelectric element mounted under each cylinder. A dynamic calibration system (San Diego Instruments, San Diego, CA, USA) was used to ensure comparable startle magnitudes. Startle stimuli were presented through a high frequency speaker located 33 cm above the startle chambers. Animals were presented with 60 startle stimuli each lasting 50 ms but with different intensities, from 70 to 120 db applied in a random order. The startle stimuli were presented with a random duration between each startle: from 5 to 20 s. Startle magnitudes were sampled every millisecond (ms) over a period of 200 ms, beginning with the onset of the startle stimulus. A startle response is defined as the peak response during 200 ms recording period.

### Fear-potentiated startle

Rats were placed in the first test chamber, a non-restrictive Plexiglas cylinder (inner diameter 8.8 cm, length 22.2 cm), the floor of which consisted of a stainless steel grid through which a software-controlled electric current could be passed. Animals were rehabituated to the startle chamber for 5 min and 5 baseline trials were administered (50-ms pulse of white noise at 120 dB) at an interstimulus interval of 30 s. The purpose of these baseline trials was to familiarize the animal with the startle stimulus in order to facilitate more accurate measurement of the animal's overall startle amplitude. Next, animals were presented with 20 light-shock pairings, at 30 s intervals. The shock (0.6 mA) was presented during the last 500 ms of the 5 s light pulse. The light stimulus was delivered via a 3-watt incandescent light bulb fastened to the inside wall of the startle chamber. After completion of the conditioning trials, animals were returned to their home cages. The same testing procedure was applied on the following day, except that 20, rather than 5, baseline trials were administered before testing. Additionally, startle measurements were made in the same grid holder that was used to condition the animals. After delivery of the final baseline trial, animals were randomly presented 10 startle stimuli, each with an intensity of 120 dB and duration of 50 ms. In half of the trials, the startle stimulus was presented concomitantly with the conditioned stimulus (CS light). Startle stimuli paired with the CS were delivered during the last 50 ms of the 5 s light presentation. The magnitude of the difference between the startle response at 120 db (Vmax) in the presence or absence of the CS will be a reflection of fear-behavior. (Walker et al., 2003).

### Open field

Animals were individually tested for 5 min each in an open field (OF) arena (43.2 × 43.2 cm) that had transparent acrylic walls and a white floor (model ENV-515, MedAssociates Inc, St. Albans, VT 05478). Each subject was initially placed in the center of the arena and horizontal activity and instant position were registered, using a system of two 16-beam infrared arrays connected to a computer. Total distances were used as indicators of locomotor activity. Times and distances in the pre-defined central and peripheral areas were recorded and used to calculate the ratio of time spent in the central area over total time of the trial, and distance travelled in the central as a function of total area. Number and duration of rearings were recorded. The test room was illuminated with bright white light.

#### Corticosterone measurement

At the end of the stress protocol (24 h after the last stressor) blood was collected for corticosterone assessment. Collection was performed at different time-points: one between 9 and 10 a.m. (starting 1 h after “lights on”) and the other between 6 and 7 p.m. (ending 1 h before “lights off”). The blood was rapidly collected after a small incision in the tail of the animals. After collection, blood was centrifuged at 13,000 rpm for 10 min. Serum (supernatant) was removed and stored at −80°C until further analysis. Corticosterone levels were measured by radioimmunoassay using a commercial kit (R&D Systems, Minneapolis, MN, USA) according to the manufacturer's instructions. Briefly, serum samples were diluted (1:200) with steroid diluent. After dilution, 100 μL of each serum sample were added to the respective tube in duplicate. To each sample, 200 μL of Corticosterone 125-I was added, immediately followed by addition of 200 μL of anti-CORT. Samples were incubated at room temperature for 2 h. After incubation, 500 μL of precipitant solution was added to all samples and then centrifuged at 2500 rpm for 15 min. CORT concentration in the precipitate was measured using an automatic gamma counter (Perkin Elmer 1470, Manchester, United Kingdom).

### Histological procedures

**Figure 1:**
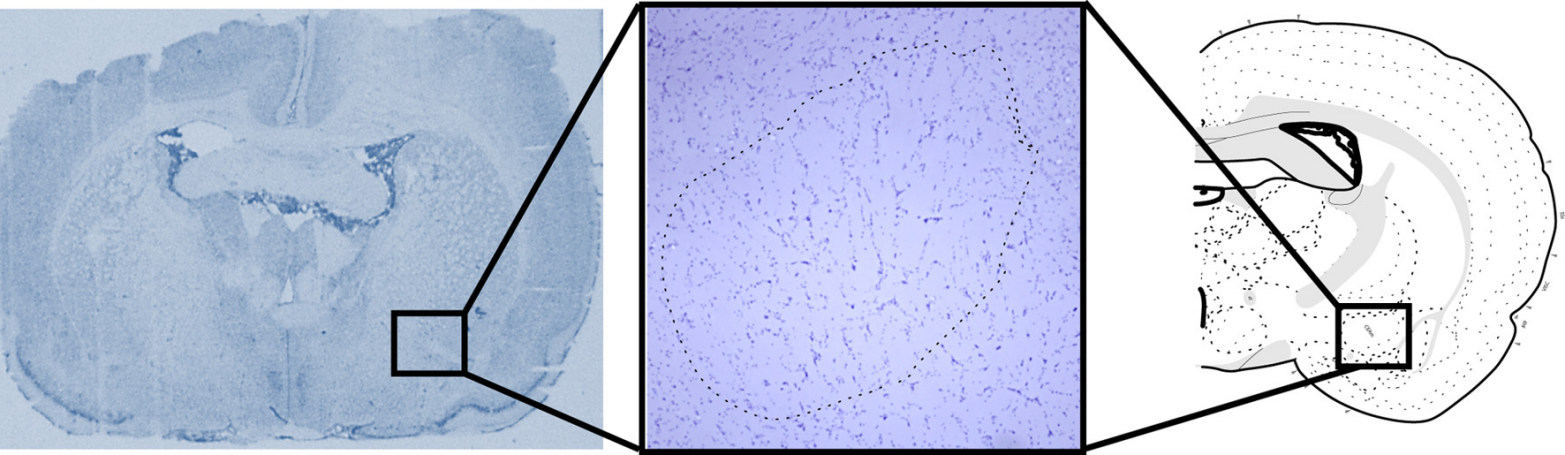
**Representative picture of the lesion sites in the Central Nucleus of the Amygdala (CeA).** Atlas section according to Swanson (1998).

Following behavioral tests the animals were deeply anaesthetized with pentobarbital and perfused transcardiacally with saline. Brains were collected, involved in Optimal Cutting-Temperature compound (Leica Biosystems, Wetzlar, Germany) and frozen. The brains were kept at −20°C until histological processed; 20 μm coronal sections were obtained in a cryostat (Leica) and stained with cresyl violet to assess the location of lesions (Figure 1).

### Statistical analysis

Results are expressed as mean ± standard error of the mean (SEM). Statistical analysis was performed using repeated measures test or Two-Way ANOVA to compare means between groups where appropriate. *Post-hoc* analysis was performed using LSD test. Statistical significance was accepted when *p* < 0.05.

## Results

**Figure 2:**
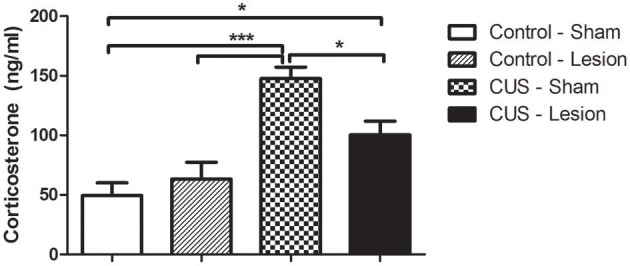
**Corticosterone levels measured in the plasma of rats collected from 9 to 10 a.m.** CUS induces an increase in corticosterone levels when comparing with controls. Interestingly, stressed animals lesioned in the CeA display an attenuation of this increase in corticosterone levels. CUS, chronic unpredictable stress; ^*^p < 0.05; ^***^p < 0.001. Results are presented as Mean + SEM.

The stress treatment induced an overall alteration in body weight gain [*F*_(1, 37)_ = 16.31; *p* < 0.001; Table 1]. Exposure to CUS protocol lead to a significant decrease in body-weight gain when compared with control groups (*p* < 0.05; Table 1) although animals with lesion in central amygdala (CUS-Lesion) had a smaller reduction in body-weight gain when compared with CUS-Sham (Table 1). Both CUS groups showed a non-significant decrease in thymus weight when compared with control animals (Table 1). CUS-Sham animals showed a non-significant increase in adrenal weight when compared with both control groups and CUS-Lesion animals (Table 1). The efficacy of the stress was also measured by assessing the corticosterone levels in the blood. The treatment induced an overall alteration of the plasma corticosterone levels 1 h after “lights on” (from 9 to 10 a.m.) [Interaction: *F*_(1, 30)_ = 6.70; *p* = 0.015] with stressed animals showing an increase in corticosterone when comparing with controls (Cont-Sham vs. CUS-Sham: *p* < 0.001; Cont-Sham vs. CUS-Lesion: *p* = 0.01; Cont-Lesion vs. CUS-Sham: *p* < 0.001). The presence of a lesion in the CeA was able to attenuate the increase of the levels of corticosterone in the plasma induced by stress (CUS-Sham vs. CUS-Lesion: *p* = 0.015) (Figure 2). There were no differences between the plasma corticosterone levels measured from 6 to 7 p.m. (data not shown).

**Figure 3:**
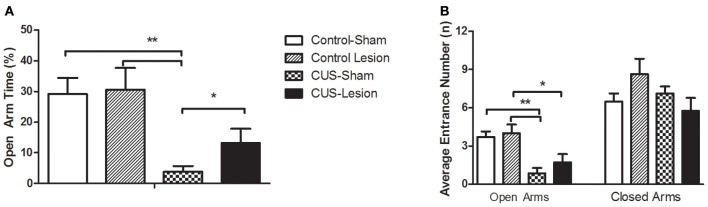
**Anxiety-like behavior measured in the Elevated Plus-Maze. (A)** Percentage of time spent in open arms. **(B)** Mean number of entrances in the open and closed arms. CUS, chronic unpredictable stress; ^*^p < 0.05; ^**^p < 0.01. Results are presented as Mean + SEM.

In the EPM, we observe differences between groups in the time spent in open arms [interaction: *F*_(1, 42)_ = 4.26; *p* = 0.044]. CUS induced anxiety-like behavior in non-lesioned animals when comparing with controls as revealed by the reduction of time spent in open arms [*F*_(1, 42)_ = 19.47; *p* < 0.01] and by the reduced number of entries in the open arm [*F*_(1, 42)_ = 7.89; *p* < 0.001]. In contrast, in CeA lesioned animals, the same CUS protocol did not induce a significant decrease in the time spent in the open arms (Cont-Lesion vs. CUS-Lesion: *p* = 0.164); importantly, the comparison amongst CUS groups show a decreased anxiety-like behavior with stressed-lesioned animals spending more time in open arms than stressed non-lesioned animals (CUS-Sham vs. CUS-Lesion *p* < 0.048). The comparison amongst control groups did not reveal an effect of the CeA lesion in this parameter (*p* = 0.44), which demonstrates that the lesion on its own is not able to induce behavioral alterations in the EPM. Furthermore, there was no significant difference between groups in the number of explorations or the number of entrances in closed arms, showing that the animals presented similar exploratory/locomotor activity [Interaction between treatments: Entrances: *F*_(1, 40)_ = 0.513, *p* = 0.68; Explorations: *F*_(1, 40)_ = 0.427, *p* = 0.74] (Figure 3).

**Figure 4:**
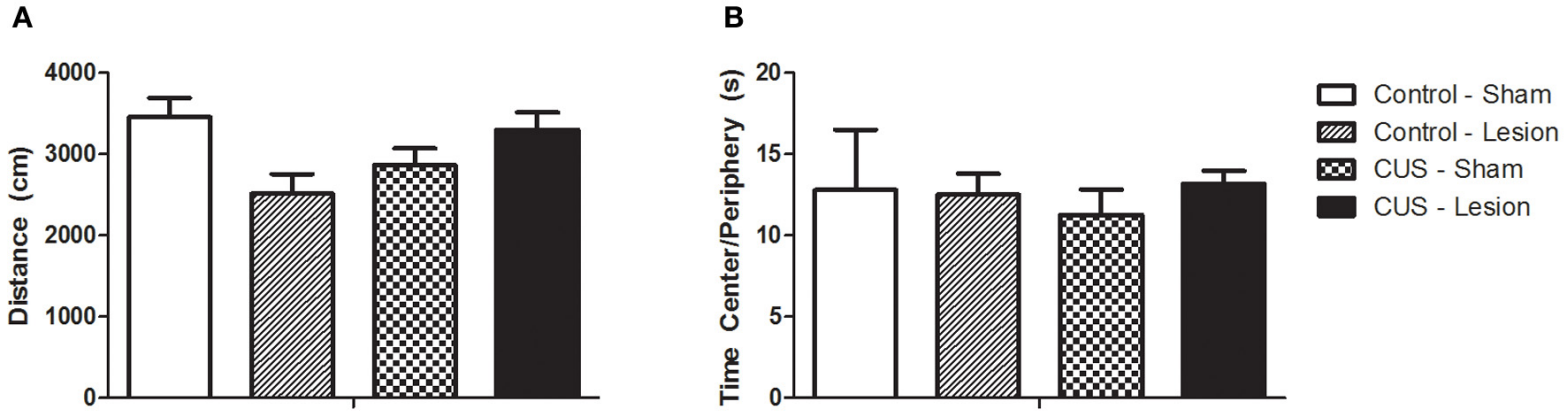
**Open field test. (A)** Total distance ran in the open field test. **(B)** Ratio between time spent in center and periphery. No statistical differences were found between groups. CUS, chronic unpredictable stress. Results are presented as Mean + SEM.

Locomotor activity was assessed with the open field test and no differences were found for the interaction between treatments or between control groups and animals submitted to CUS protocol [*F*_(1, 42)_ = 1.07, *p* = 0.37]. Furthermore, there were no significant differences between groups in the time spent in center/peripheral areas of the open-field [*F*_(1, 42)_ = 0.18, *p* = 0.68] or in the number of rearings and in the time spent in rearing activity, indicating no alterations in exploratory activity [*F*_(1, 42)_ = 1.87, *p* = 0.46] (Figure 4).

**Figure 5:**
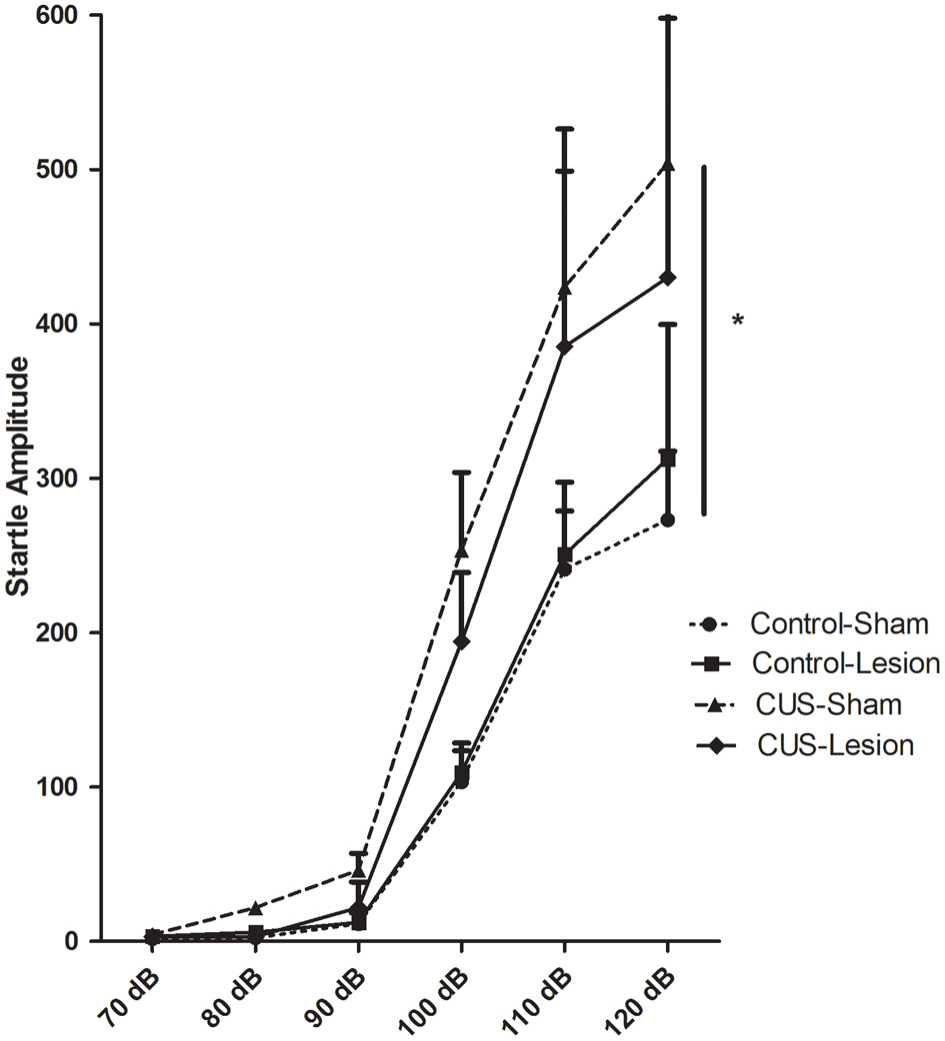
**Acoustic startle responses.** Startle amplitude in response to an acoustic stimulus. CUS induces an increase in the startle when comparing with controls. CUS, chronic unpredictable stress; ^*^p < 0.05. Results are presented as mean ± SEM.

In the acoustic startle test there is an overall increase in responsiveness to the stimuli with the experimental procedures [*F*_(1, 32)_ = 3.99, *p* = 0.02]. Despite a trend for an attenuation in the responsiveness to the startle in stressed animals injected with ibotenic acid (CUS-Lesion) when compared with CUS-Sham group, this difference did not reach statistical significance (at 120 db: *p* = 0.16). There was also no significant difference between both control groups (Figure 5). These results suggest that a lesion in the CeA may attenuate the stress effects in this reflex response.

**Figure 6:**
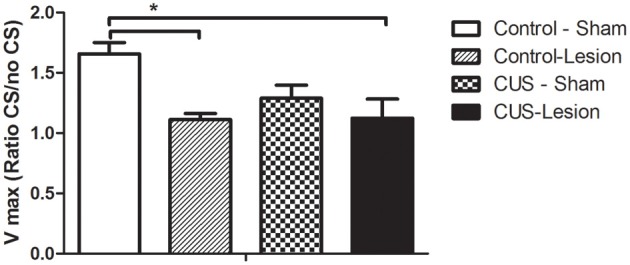
**Fear-potentiated startle responses.** Startle amplitude in response to an acoustic stimulus. No statistical differences were found between groups. CS, conditioned stimulus; CUS, chronic unpredictable stress rats. Vmax—startle amplitude at 120 db. Results are presented as mean ± SEM. ^*^p < 0.05.

We have also evaluated the fear-potentiated startle of these animals and found that the interaction between stress and lesion does not induce any significant differences [*F*_(1, 21)_ = 2.74; *p* = 0.12]. Nevertheless, animals lesioned in the CeA displayed significant alterations in startle behavior [*F*_(1, 21)_ = 9.64; *p* = 0.061] when compared with non-lesioned animals. (Figure 6).

## Discussion

Herein, we show for the first time that excitotoxic lesions in the CeA attenuate stress-induced anxiety behavior and also attenuated the activation of the HPA axis. Previous studies have demonstrated that lesions in CeA affect the manifestation of fear-behavior but not light-enhanced startle, a behavior more associated with a display of anxiety (Walker and Davis, 2008; Davis et al., 2010). In fact, it is widely accepted that while amygdala is essential for the manifestation of fear-behavior, the BNST is more determinant for anxiety-like behavior (Walker et al., 2003; Hammack et al., 2004; Lee et al., 2008). It is important to note that the attenuation of the stress-induced anxiety in the CeA lesioned animals was observed under stress conditions but it did not induce any alteration in anxiety-like behavior in control animals. In other words, it demonstrates that the integrity of the CeA is required for the establishment of stress-induced hyperanxiety but does not mediate anxiety behavior *per se*. Similarly, CeA lesions were able to blunt the increase in circulating corticosteroids induced by stress exposure while not altering the levels of corticosteroids in control animals. In accordance, previous reports have shown (Prewitt and Herman, 1997) that animals that were lesioned in CeA show impaired activation of the HPA axis after stress, as well as no alterations in adrenals and thymus weight when compared with controls.

It is important to highlight that the attenuation of stress-induced anxiety is not complete, which suggests that other pathways are still conveying the changes in the neuronal networks that rule stress-induced anxiety behavior. This is not surprising if one considers that there are several factors contributing to the activity of anxiety circuits, namely at BNST level. Of relevance, the anterior BNST receives a strong glutamatergic projection from the infralimbic cortex that will likely contribute to anxiety-like behavior (Sesack et al., 1989; Hurley et al., 1991; Massi et al., 2008). Surprisingly, differences in anxiety behavior were not coincident among behavioral tests. While CeA lesions attenuated anxiety-like behavior in the EPM we only found a non-significant trend in the acoustic startle response. Additionally, no differences were found in the open field test between control and CUS animals or any effect of lesions. In fact, these results were already described by our group (Pêgo et al., 2008). This apparent discrepancy may reflect specificity of the effect of treatment and/or lesion on these behavioral tasks. While all behavioral tasks are affected by the baseline level of anxiety of the individual, the EPM additionally reflects a decision-making process that involves cortical processing and assessment. Cortical functions are known to be affected by CUS (Cerqueira et al., 2007) and the specificity of the observed changes in the EPM may reflect this particular sensitivity of upstream regulatory areas like the prefontal cortex to stress. Reflex behavior, which is not mediated by the amygdala (Koch and Schnitzler, 1997) or pure exploratory behavior as observed in the open field is unlikely to be affected by cortical functions and this may justify the results reported.

In contrast, we confirmed that a fully functional CeA is essential for the manifestation of fear behavior, measured in the fear-potentiated startle. In fact, a lesion of CeA, triggered a disruption of fear behavior in both stressed and non-stressed animals. Moreover, and also in accordance with our previous reports (Pêgo et al., 2008), the CUS protocol did not induce an alteration in the fear-potentiated behavior. These facts demonstrate that the contribution of the main output of the amygdala (CeA) is quite distinct in these behaviors: while it is determinant for the fear-potentiated startle in non-stressed conditions, the effect in anxiety-like behavior is only present when this behavior is being triggered by a complementary insult (in the present case, stress). This is not surprising when considering that the neuronal circuits involved in fear-potentiated startle critically depend on the projection of the CeA to the caudal pontine reticular nucleus (Davis et al., 2010), whereas for anxiety behavior, and for the control of HPA activity, the output of the CeA to the BNST represent only one of the possible modulators of its activity.

Given the topographical organization of the projections of the CeA, it is likely that an excitotoxic lesion in CeA will lead to a reduction of GABA and peptidergic inputs, particularly CRF, into the anterior BNST (Veinante et al., 1997; Day et al., 1999). Taking into account that the increase in CRF following chronic stress has a fundamental role in the activation of anterior BNST and consequent activation of the HPA axis (Ventura-Silva et al., 2012), this might be one plausible explanation for the attenuation of the stress-induced anxiety behavior in CeA lesioned animals. Of relevance, a lesion in the anterior BNST induces an attenuation of the activity of the HPA axis in a basal situation although no alterations in animals submitted to chronic variable stress (Choi et al., 2008a) in opposition to the posterior BNST that contributes to the inactivation of the HPA axis (Choi et al., 2008b). These observations show that the anterior BNST seems to be essential for the regulation of the HPA axis in a basal situation and together with our observations, CeA contributes to the manifestation of a stress-related phenotype.

In further support of this hypothesis is the fact that a lesion in CeA did not affect anxiety-behavior in baseline conditions, which is in line with previous observations by other labs (Möller et al., 1997; McHugh et al., 2004; Cai et al., 2012). Our results are consistent with reports showing that the manipulation of CeA can affect the expression of anxiety-like behavior. In particular, lentiviral overexpression of CRF in the CeA results in the dysregulation of the hypothalamic-pituitary-adrenal (HPA) axis and alterations in the baseline response to acoustic stimuli (Keen-Rhinehart et al., 2009).

In summary, we observed that, in control animals, a lesion in CeA triggers an alteration in the fear-potentiated startle but not in anxiety-like behavior. Nevertheless, when animals are submitted to a chronic stress protocol the CeA lesion partially attenuates the development of anxiety-like behavior. These findings contribute to better understand the role of the CeA in the pathogenesis of anxiety and fear behavior and in the sense to know how its modulation might be of relevance for the control of emotional disturbances involving such behaviors but also for the control of HPA activity.

### Conflict of interest statement

The authors declare that the research was conducted in the absence of any commercial or financial relationships that could be construed as a potential conflict of interest.

## Abbreviations

BNST: Bed Nucleus of the Stria Terminalis
CeA: Central Nucleus of the Amygdala
Cont: Control
CRF: Corticotrophin Releasing Factor
CUS: Chronic Unpredictable Stress
EPM: Elevated-Plus Maze
HPA: Hypothalamus-Pituitary-Adrenals
PBS: Phosphate buffer solution
PVN: Paraventricular Nucleus of the Hypothalamus
SEM: Standard Error of the Mean.
